# Feedback from group III/IV afferents in skeletal muscle slows heart rate acceleration when transitioning from rest to low‐intensity exercise in males

**DOI:** 10.1113/EP093776

**Published:** 2026-07-11

**Authors:** Shane Burgess, Simranjit K Sidhu, Samuel Chalmers, Markus Amann, Jonathan D Buckley

**Affiliations:** ^1^ Alliance for Research in Exercise, Nutrition and Activity (ARENA), School of Allied Health and Human Performance Adelaide University Adelaide South Australia Australia; ^2^ Department of Physiotherapy James Cook University Townsville Queensland Australia; ^3^ School of Pharmacy and Biomedical Sciences Adelaide University Adelaide South Australia Australia; ^4^ Department of Anesthesiology University of Utah Salt Lake City Utah USA

**Keywords:** cycling exercise, heart rate kinetics, intrathecal fentanyl, maximal rate of heart rate increase

## Abstract

Group III/IV skeletal muscle afferents respond to intramuscular mechanical and metabolic stimuli and are more active in fatigued muscle. These afferents project onto the brainstem and motor cortex and modulate cardiovascular and motor responses during exercise. The maximal rate of heart rate (HR) increase (rHRI), a marker of HR acceleration during the rest‐to‐exercise transition, is linearly related to exercise performance across various training and fatigue states, being faster with better exercise performance and vice versa. This study evaluated the effect of feedback from group III/IV afferents on rHRI in non‐fatigued muscle. HR data were collected in eight recreationally active male participants (24 ± 1 years) during the transition from rest to 3 min of cycling at 100 W under control (CON) conditions and following lumbar intrathecal fentanyl (FENT) infusion to impair feedback from group III/IV afferents of the leg. rHRI was defined as the maximal first‐derivative value of a logistic curve fitted to the HR data. Pre‐exercise HR was similar in CON and FENT (CON 91.7 ± 16.7 bpm, FENT 99.8 ± 15.5 bpm, *P* = 0.250). HR increased more with exercise during CON than FENT (B = 10.01, 95% CI 5.33 to 14.70, *P *< 0.001) but rHRI was faster during FENT (B = 1.52, 95% CI 0.24 to 2.80, *P* = 0.020). In non‐fatigued muscle, feedback from group III/IV afferents slowed rHRI. Increased feedback when muscle is fatigued may slow rHRI further which may explain the relationship between rHRI and exercise performance seen in previous studies, but further studies on fatigued muscle are required to confirm this.

## Abstract (highlights)

- **What is the central question of this study?**
  How does feedback from group III/IV skeletal muscle afferents influence heart rate acceleration during the transition from rest to light exercise in non‐fatigued muscle?
- **What is the main finding and its importance?**
  Feedback from group III/IV skeletal muscle afferents slowed heart rate acceleration at exercise onset. Because group III/IV afferents are more active in fatigued muscle and inhibit motor output, contributing to central fatigue, they may further reduce heart rate acceleration under fatigued conditions and link slowed heart rate acceleration with reduced exercise performance, and vice versa, which has been observed in previous studies.

## 1 INTRODUCTION

The increase in heart rate (HR) during the transition from rest to exercise is due primarily to initial withdrawal of parasympathetic activity followed by increasing sympathetic activity as exercise intensity increases (White & Raven, 2014). At the onset of exercise somatomotor centres in the motor cortex and autonomic nuclei in the cardiovascular centre in the brainstem are activated simultaneously and comprise a feed‐forward pathway known as central command (Fisher, 2014). Central command couples skeletal muscle contraction with changes in parasympathetic and sympathetic modulation of cardiovascular responses, including increasing HR at the onset of exercise (Fisher, 2014). In addition to the feedforward central command pathway, feedback pathways (baroreflex, chemoreflex and exercise pressor reflex) also contribute to the modulation of cardiac autonomic adjustments during exercise through modulation of parasympathetic and sympathetic activity (Fisher et al., 2015).

The maximal rate of HR increase (rHRI) is a marker of HR acceleration during the transition from rest to standardised light‐intensity exercise. rHRI has been assessed immediately prior to performing maximal aerobic and anaerobic exercise across different phases of training and states of acute fatigue (D'Unienville N et al., 2022; Thomson et al., 2015) and fatigue induced from chronic overload training (Nelson et al., 2020). This has resulted in linear relationships being identified between rHRI and maximal aerobic (Nelson et al., 2014, 2017, 2020; Thomson et al., 2015) and anaerobic (D'Unienville N et al., 2022) exercise performance in both males (Nelson et al., 2014, 2020) and females (D'Unienville N et al., 2022; Nelson et al., 2017). In these studies, endurance exercise performance was defined as the amount of work done during a cycling time‐trial or time taken to run a set distance on a treadmill, and anaerobic exercise performance was defined as the peak power output during a maximal cycle sprint and/or the amount of work done during a 30 s maximal effort on a cycle ergometer. Specifically, these studies showed that rHRI was faster when exercise performance was improved and slower when exercise performance was impaired. These findings led to it being suggested that rHRI could be used as a submaximal assessment to monitor how athletes are responding to training to inform changes in training loads to optimise performance (D'Unienville N et al., 2022; Nelson et al., 2020; Thomson et al., 2015).

Group III/IV skeletal muscle afferents respond to changes in the intramuscular mechanical and metabolic environment (Adreani et al., 1997). These afferents project to central neural circuits in the brainstem and changes in their activity alter HR via changes in sympathetic and parasympathetic modulation (Kaufman et al., 2002), typically promoting sympathetic activity and inhibiting parasympathetic activity (Fisher, 2014). These afferents also project to the motor cortex and as muscle becomes fatigued their output increases, inhibiting the excitability of motor neurons and contributing to the development of central fatigue (Taylor et al., 2016). This effect of feedback from these afferents on modulation of both motor output and HR has led to it being proposed (D'Unienville N et al., 2022; Thomson et al., 2015) that feedback from these afferents might underpin the relationship between changes in rHRI and changes in exercise performance that have been observed in previous studies (D'Unienville N et al., 2022; Nelson et al., 2014, 2017, 2020; Thomson et al., 2015). In essence, if signalling from group III/IV skeletal muscle afferents inhibits rHRI, then the heightened activity of these afferents during muscle fatigue would be expected to slow rHRI as fatigue progressively increases. At the same time increased signalling from these neurons in fatigued muscle would also be expected to inhibit motor output. This would potentially explain the slowing of rHRI in concert with reduced motor output and reduced exercise performance and vice versa that has been observed in previous studies (D'Unienville N et al., 2022; Nelson et al., 2014, 2017, 2020; Thomson et al., 2015).

The current investigation was based on data collected in a previous study (Sidhu et al., 2018) that evaluated the effect of group III/IV afferents on corticospinal excitability. That study used lumbar intrathecal infusion of fentanyl to inhibit feedback from group III/IV leg muscle afferents and HR data were collected as R‐R intervals during cycling at 100 W. Those data enabled calculation of rHRI and evaluation of the effect of feedback from group III/IV locomotor muscle afferents on rHRI during non‐fatiguing exercise, which was the aim of the present study. It was hypothesised that feedback from group III/IV leg muscle afferents would slow rHRI.

## 2 METHODS

### 2.1 Ethical approval

This study comprised a secondary analysis of data collected as part of another study (Sidhu et al., 2018). All experimental procedures for the primary study were approved by the University of Utah and Salt Lake City Veterans Affairs Medical Center Institutional Review Boards (IRB approval number: 62914). The study conformed to the standards set by the *Declaration of Helsinki*, except for registration in a database. All participants provided written informed consent prior to participation. The secondary analysis of data for the present study was approved by the Human Research Ethics Committee of the University of South Australia (now renamed as Adelaide University – approval number: 205147).

### 2.2 Study design

In brief, the components of the primary study that were relevant for the present analysis were that the primary study used a randomised, counter‐balanced, cross‐over design in which HR was measured during rest, followed by 3 min of cycling at 100 W, on two separate occasions over two successive weeks. On one occasion data were collected under control conditions (CON) and on the other occasion after lumbar intrathecal infusion of fentanyl through the L3–4 intervertebral space (FENT) to inhibit feedback from group III/IV skeletal muscle afferents from the leg muscles.

### 2.3 Participants

HR data for eight male participants tested in the primary study were available for the present analysis. Of the 11 original participants (9 male, 2 female) data for one male and both female participants were lost because of technical difficulties with HR recording. Therefore, it was not possible to include any data for female participants in the present analysis. The eight male participants for whom data were available were 24 ± 1 years of age with a body mass of 76 ± 4 kg, were 1.7 ± 0.2 m in height and had a maximal oxygen uptake of 45 ± 3 mL/kg/min.

### 2.4 Experimental protocol

The component of the primary study protocol that is relevant for the present study involved participants being seated on a cycle ergometer (Velotron, Elite Model, Racer Mate, Seattle, WA, USA) while HR was recorded as R‐R intervals using a 12‐lead electrocardiogram (Nasiff Cardiocard, Central Square, NY, USA) prior to and during 3 min of cycling at 100 W. All participant testing sessions were performed at the same time of day.

Various protocols utilising a range of exercise intensities and durations during the assessment of rHRI have been evaluated to identify the protocol which provided the strongest relationship between rHRI and exercise performance. Ultimately, the strongest and most consistent relationships between rHRI and exercise performance were identified when rHRI was assessed during the transition from rest to a constant low exercise intensity (e.g. cycling at 100 or 120 W; Bellenger et al., 2017; D'Unienville N et al., 2022; Nelson et al., 2017) for a duration of 3 min (Nelson et al., 2020). Hence, the data available from the primary study which required participants to cycle at 100 W for 3 min were considered suitable for the assessment of rHRI.

To determine rHRI, HR versus time (*t*) was modelled using a sigmoidal logistic curve (Equation 1) fit in MATLAB (version R2019b,The Math Works Inc., Natick, MA, USA) to 20 s of resting HR data immediately preceding the commencement of exercise and 3 min of constant load (100 W) cycling exercise HR data. We have previously established that use of a sigmoidal curve fit is superior to other curves (i.e. single exponential curve fit) for determining rHRI (Thomson et al., 2015).

(1) $$\mathrm{HR}=C3/(1+\mathrm{ex}p^{-C1\left(t\;-\;C2\right)})$$

In Equation 1, C1 represents the logistic growth rate, C2 is the time (s) at the midpoint of the curve, and C3 is the ΔHR from pre‐exercise to the steady‐state HR at 100 W. rHRI was estimated as the first derivative maximum of the curve using Equation 2:

(2) rHRI=(C1×C3)/4

The goodness of fit for the mathematical modelling of rHRI was assessed using the coefficient of determination (*r* ^2^) and mean square error (MSE).

### 2.5 Statistical analysis

**FIGURE 1:**
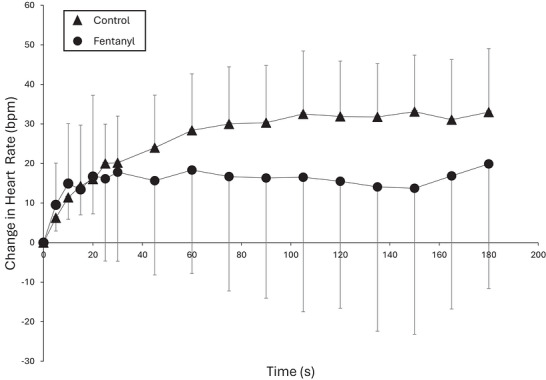
Increase in heart rate during 3 min of cycling at 100 W under control conditions and following intrathecal infusion of fentanyl. Significantly greater increase in heart rate in Control vs. Fentanyl (*P* < 0.001). Data represent means ± standard error. Data were missing for three participants (*n* = 2 Control, *n* = 1 Fentanyl) resulting in data being available for *n* = 6 participants under control conditions and *n* = 7 with fentanyl. All six participants had 13 data points available under control conditions (total 78 data points). With fentanyl five participants had all 13 data points available, one had eight data points available (0 to 105 s) and one had six data points available (0 to 75 s) (total 79 data points available). (Raw data provided in Supplementary Information).

Statistical analysis was performed using Stata/IC 16.0 (StataCorp, College Station, TX, USA). Seven CON and eight FENT HR data files were available for analysis from the eight participants. However, due to substantial artefacts in two HR data files (one CON and one FENT), these files could not be modelled to determine rHRI. Therefore, six CON and seven FENT data files from the eight participants were modelled. A random‐effect mixed model was used to evaluate if there were differences between FENT and CON. Treatment (FENT, CON) was included as a fixed effect and each participant was included as a random effect in the model. Likelihood tests were performed to ensure the data were normally distributed. The advantage of using a mixed‐model for this analysis was that mixed‐model analysis does not require a complete data set, allowing all available data collected from the participants to be used in the analysis. This avoids bias that can be introduced by excluding participants who do not have complete data available (Gabrio et al., 2022). Data are reported as B‐coefficients, 95% confidence intervals and *P*‐values. Values are represented as means ± standard deviation except for Figure 1 where the purpose was to represent differences in mean HR responses at various time points during exercise rather than variance in responses between FENT and CON so data are presented as means ± standard error of the mean. Statistical significance was set at an α‐level of 0.05.

## 3 RESULTS

**FIGURE 2:**
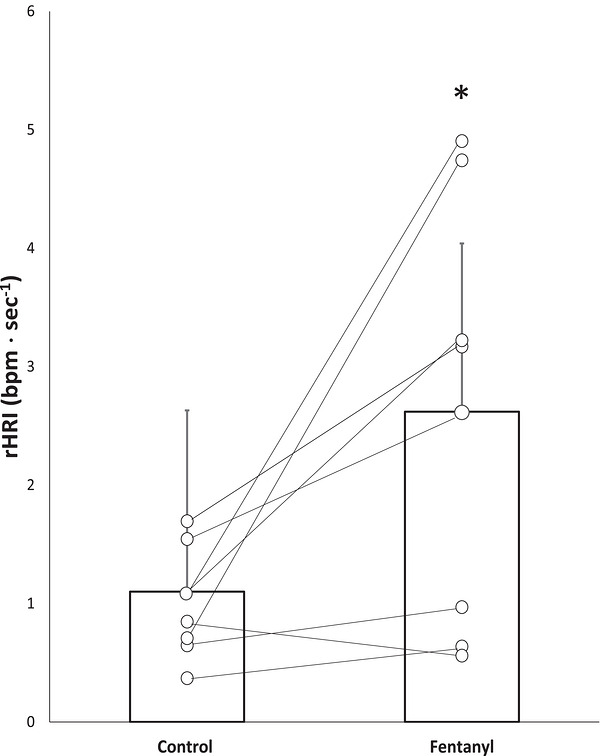
Maximal rate of increase in heart rate (rHRI) during the transition from rest to 3 min of cycling at 100 W following lumbar intrathecal infusion of fentanyl or control. Values are estimated marginal means ± standard deviation. Missing data points (*n* = 2 Control, *n* = 1 Fentanyl) have been replaced with the estimated marginal mean for graphical purposes, providing data for *n* = 8 participants in total for each condition. *Significantly different from control (*P* = 0.020).

Pre‐exercise HR was not different between FENT and CON (CON 91.7 ± 16.7 bpm, FENT 99.8 ± 15.5 bpm, *P* = 0.250). HR increased more in CON compared to FENT during the 3 min of cycling (B = 10.01, 95% CI 5.33 to 14.70, *P* < 0.001); Figure 1). However, despite the lesser increase in HR during the cycling exercise with FENT compared with CON, rHRI was faster with FENT compared to CON (B = 1.52, 95% CI = 0.24 to 2.80, *P* = 0.020; Figure 2).

The goodness of fit for the modelling of rHRI provided an *r* ^2^ of 0.41 ± 0.25 and MSE of 119.80 ± 85.07.

## 4 DISCUSSION

The main finding of this study was that during the transition from rest to light non‐fatiguing cycling exercise feedback from group III/IV leg muscle afferents slowed rHRI. As muscle fatigues, the output of these group III/IV afferents increases, which inhibits motor output and contributes to the development of central fatigue (Sidhu et al., 2018). If this greater afferent activity in fatigued skeletal muscle also slows rHRI, it could explain why a slower rHRI has been associated with impaired exercise performance in previous studies, and vice versa (D'Unienville N et al., 2022; Nelson et al., 2014, 2017, 2020; Thomson et al., 2015).

It is well established that, at the onset of exercise the combination of central command and feedback from group II/IV afferents promotes withdrawal of parasympathetic modulation which results in the initial increase in HR during light‐intensity exercise, such as was performed in the present study (Nakamura et al., 1993; Robinson et al., 1966; Wan et al., 2023), with further increases in HR resulting from increased sympathetic modulation as the exercise intensity progresses to moderate–high intensity (Nakamura et al., 1993; Robinson et al., 1966; Wan et al., 2023). The finding of the present study, and that of a previous study (Amann et al., 2010), that steady‐state HR increased less during cycling exercise at 100 W when feedback from group III/IV afferents was inhibited with fentanyl, confirms that feedback from these afferents increases steady‐state HR during exercise. However, while steady‐state HR increased less during exercise with FENT, rHRI was, perhaps counterintuitively, faster. At the low exercise intensity (100 W) at which rHRI was evaluated in the present study, HR would have increased primarily through the withdrawal of parasympathetic activity. The faster rHRI with blocked muscle afferents suggests that feedforward processes from central command, which would have been unaffected by fentanyl administration, may act to facilitate very rapid parasympathetic withdrawal, while feedback from group III/IV afferents may act to promote slower parasympathetic withdrawal, at least at a light exercise intensity such as the 100 W used here. Thus, the actual rate of parasympathetic withdrawal, and accordingly the rate of HR acceleration, may reflect a balance between inputs from central command, which promote more rapid withdrawal, and feedback from group III/IV muscle afferents, which promotes slower withdrawal.

### 4.1 Limitations and practical implications

A limitation of the present study was that while feedback from group III/IV afferents may modulate HR and rHRI via the pressor reflex, it is not the only contributor to HR regulation during exercise. HR responses to exercise are also modulated by the baroreflex to maintain blood pressure (Wan et al., 2023) and, while it was not measured in the present study, increases in mean arterial blood pressure during exercise have been shown to be partially attenuated with fentanyl blockade (Amann et al., 2010; Hureau et al., 2018). This attenuation is due to inhibition of feedback from group III/IV afferents which, under control conditions, resets the baroreflex operating point so it operates at higher transmural pressures during exercise (Hureau et al., 2018). A resetting of the baroreflex operating point at a lower level during exercise with fentanyl would have contributed to the lower steady‐state HR that was observed, but it is not clear whether it would have had an effect on increasing rHRI during the transition from rest to exercise given that while feedback from group III/IV afferents is critical for resetting the carotid baroreflex and heart rate operating points during exercise, it is not involved in spontaneous baroreflex responsiveness, so is not involved in moment‐to‐moment regulation of blood pressure and HR (Hureau et al., 2018).

Furthermore, the design of the primary study from which the data for the present analysis were derived involved three forms of stimulation that were applied during the cycle exercise at 100 W when rHRI was determined (magnetic transcranial stimulation and electrical motor nerve and cervicomedullary stimulation). It is possible that these stimulations may have influenced the HR response to exercise (Maharjan et al., 2022; Paxton et al., 2011; Schmausser et al., 2022), but they were given at the same times and same intensities under both the CON and FENT conditions such that any effect on HR or rHRI should have been consistent across conditions and therefore to some extent have been controlled for.

A final limitation is that data were only available for male participants from the primary study and, given there are sex‐related differences in the autonomic control of HR during exercise (Kim et al., 2012; Wan et al., 2022), the findings of the present study may not be applicable to females. However, linear relationships have been reported between rHRI and exercise performance in both males and females.

In conclusion, it appears that feedback from group III/IV afferents slows the acceleration of HR during the transition from rest to light‐intensity exercise. However, whether this feedback modulates the relationship between rHRI and exercise performance that has been reported in previous studies remains unclear and will require similar studies to the present one to be performed in individuals under differing states of training and fatigue.

## AUTHOR CONTRIBUTIONS

Simranjit K. Sidhu and Markus Amann conceived and performed the primary study and Simranjit K. Sidhu performed the experiments. Jonathan D. Buckley, Shane Burgess and Samuel Chalmers conceived and designed the secondary analysis that comprised the present study. Shane Burgess and Jonathan D. Buckley analysed data. All authors interpreted the results. Shane Burgess drafted the manuscript and prepared figures. All authors edited and revised the manuscript. All authors have read and approved the final version of this manuscript and agree to be accountable for all aspects of the work in ensuring that questions related to the accuracy or integrity of any part of the work are appropriately investigated and resolved. All persons designated as authors qualify for authorship, and all those who qualify for authorship are listed. Current addresses of authors are as indicated in the affiliations.

## CONFLICT OF INTEREST

Jonathan D. Buckley invented the rHRI technology and it has been patented by Adelaide University. Jonathan D. Buckley is employed by Adelaide University but has assigned his rights in the patent to the University. Adelaide University has licenced the rights to commercialise the rHRI technology to a commercial partner. Samuel Chalmers is also an employee of Adelaide University. Shane Burges was supported by an Australian Postgraduate Award scholarship from the Australian Commonwealth Government. Markus Amann and Simranjit K. Sidhu declare no competing interests.

## GENERATIVE AI STATEMENT

The authors confirm that no artificial intelligence tools, including large language models (LLMs), were used in the drafting or revision of this manuscript. All content was conceived, written and approved solely by the authors.

## Supporting information



Supporting Information

## Data Availability

The data used for this study will be made available upon reasonable request.
