# Supplementary figures and images for: Feedback from group III/IV afferents in skeletal muscle slows heart rate acceleration when transitioning from rest to low‐intensity exercise in males
Source: Exp Physiol. 2026 Jul 11:10.1113/EP093776. Online ahead of print. doi: 10.1113/EP093776 (PMC13394101; doi:10.1113/EP093776)

## Slide 1
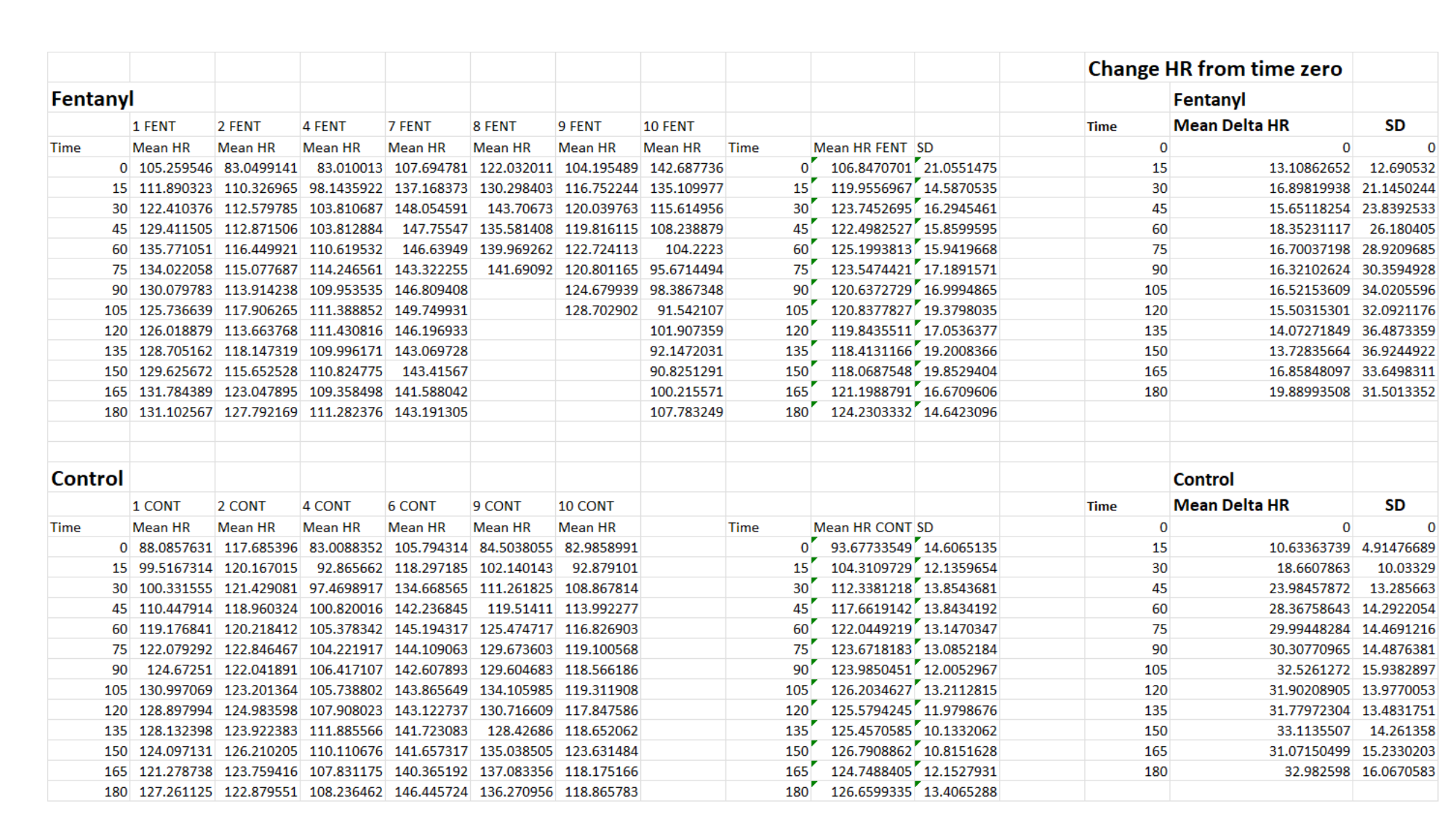

Supplement: Supplementary file 1 — Supporting Information [file EPH-9999-0-s001.pptx]
